# Parallel evolution of conserved non-coding elements that target a common set of developmental regulatory genes from worms to humans

**DOI:** 10.1186/gb-2007-8-2-r15

**Published:** 2007-02-02

**Authors:** Tanya Vavouri, Klaudia Walter, Walter R Gilks, Ben Lehner, Greg Elgar

**Affiliations:** 1 Wellcome Trust Sanger Institute, Wellcome Trust Genome Campus, Hinxton, Cambridge CB10 1SA, UK; 2 School of Biological and Chemical Sciences, Queen Mary, University of London, London E1 4NS, UK; 3 MRC Biostatistics Unit, Institute of Public Health, Cambridge CB2 2SR, UK; 4 Department of Statistics, University of Leeds, Leeds LS2 9JT, UK; 5 EMBL/CRG Systems Biology Unit, Centre for Genomic Regulation (CRG), UPF, C/Dr. Aiguader 88, Barcelona 08003, Spain

## Abstract

**Background:**

The human genome contains thousands of non-coding sequences that are often more conserved between vertebrate species than protein-coding exons. These highly conserved non-coding elements (CNEs) are associated with genes that coordinate development, and have been proposed to act as transcriptional enhancers. Despite their extreme sequence conservation in vertebrates, sequences homologous to CNEs have not been identified in invertebrates.

**Results:**

Here we report that nematode genomes contain an alternative set of CNEs that share sequence characteristics, but not identity, with their vertebrate counterparts. CNEs thus represent a very unusual class of sequences that are extremely conserved within specific animal lineages yet are highly divergent between lineages. Nematode CNEs are also associated with developmental regulatory genes, and include well-characterized enhancers and transcription factor binding sites, supporting the proposed function of CNEs as *cis*-regulatory elements. Most remarkably, 40 of 156 human CNE-associated genes with invertebrate orthologs are also associated with CNEs in both worms and flies.

**Conclusion:**

A core set of genes that regulate development is associated with CNEs across three animal groups (worms, flies and vertebrates). We propose that these CNEs reflect the parallel evolution of alternative enhancers for a common set of developmental regulatory genes in different animal groups. This 're-wiring' of gene regulatory networks containing key developmental coordinators was probably a driving force during the evolution of animal body plans. CNEs may, therefore, represent the genomic traces of these 'hard-wired' core gene regulatory networks that specify the development of each alternative animal body plan.

## Abstract 2

Invertebrate conserved noncoding elements (CNEs) are associated with the same core set of genes as vertebrate CNEs, and may reflect the parallel evolution of enhancers in the gene regulatory networks that define alternative animal body plans.

## Background

Comparisons of the human genome against the genomes of distantly related vertebrates have revealed an abundance of highly conserved non-coding elements (CNEs) that appear to have been 'frozen' throughout vertebrate evolution [1-7]. The exact number of elements shared between any set of species varies depending on the precise definition of similarity and the divergence of the genomes used. For example, a comparison of the human genome against the mouse and the rat genomes revealed that all three share 256 elements with no evidence of transcription that are 100% identical over at least 200 base-pairs (bp) [2]. Furthermore, the human genome and the genome of the Japanese pufferfish (*Fugu rubripes*), which diverged from a common ancestor approximately 450 million years ago (MYA), share 1,373 CNEs, with an average length of 199 bp and average identity of 84% [4].

A striking property of human CNEs is that they cluster in genomic regions that contain genes coding for transcription factors and signaling genes involved in the regulation of development ('trans-dev' genes) [2-4,6]. Therefore, CNEs have been proposed to act as *cis*-regulatory sequences for these trans-dev genes. In support of this, where tested, the majority of assayed CNEs can act as tissue-specific enhancers for a transgene in zebrafish or mice [4,7-10].

Vertebrate CNEs show extreme sequence conservation among distantly related species, often showing higher conservation than protein-coding exons [4,5]. However, there appear to be no traces of vertebrate CNEs in invertebrate genomes that can be identified by sequence similarity searches [2,4,11]. The evolutionary origin of most vertebrate CNEs therefore remains unknown [11]. Although CNEs have also been identified in invertebrate genomes [12-14], they have been found to be smaller and less frequent than vertebrate CNEs. Recently, Glazov *et al*. [13] identified 20,301 non-coding elements that are conserved over at least 50 bp between the very closely related genomes of *Drosophila melanogaster *and *Drosophila pseudoobscura *and showed that these elements were also found preferentially near genes encoding transcription factors and developmental regulatory genes. *D. melanogaster *and *D. pseudoobscura *diverged from their common ancestor 25 to 55 MYA [15] and show sequence divergence similar to that between the human and mouse genomes [13]. Consequently, it is difficult to distinguish functionally conserved elements from background sequence conservation by comparing these two genomes alone. In fact, only two of these elements were conserved in the more distantly related genome of *Anopheles gambiae*, which shared a common ancestor with the *Drosophila *species approximately 250 MYA [16]. Therefore, it is still unclear how widespread highly conserved non-coding elements are among different animal genomes and whether similar genes are associated with the most conserved non-coding elements in both invertebrate and vertebrate genomes.

To provide further insight into the function and evolution of CNEs, we have focused on the simplest animal group for which multiple genome sequences are currently available. Two nematode genomes, *Caenorhabditis elegans *and *Caenorhabditis briggsae *have been fully sequenced and assembled [17,18]. These two species diverged from a common ancestor approximately 80 to 110 MYA [18]. Although *C. elegans *and *C. briggsae *diverged at a similar time as human and mouse, the neutral substitution rate estimated for these two *Caenorhabditis *genomes is roughly three-fold higher than for human-mouse [18], so providing a substantial period of evolutionarily divergence between these species. Whole genome shotgun sequence has also been released recently for a third nematode genome, *C. remanei*. *C. remanei *is a sister species of *C. briggsae*, and these two genomes show sequence divergence similar to that between the human and mouse genomes [19].

The CNEs that we have identified in *C. elegans *have many properties that mirror those of vertebrate CNEs. Although smaller than vertebrate CNEs, worm CNEs also reside near developmental regulatory genes. Moreover, they share both a striking base composition transition signal and a similar A+T content with vertebrate CNEs. Worm CNEs identify many previously characterized transcriptional enhancers and transcription factor binding sites. Most strikingly, we find that vertebrate and invertebrate CNEs are often associated with orthologous genes. Our analysis indicates that CNEs are commonly associated with the same developmental genes in different animal groups. Therefore, it seems likely that CNEs evolved in parallel in different animal lineages to regulate the expression of a core set of regulatory genes. The extreme sequence conservation of CNEs likely reflects the functional importance of these elements as components of the gene regulatory networks that define each different evolutionarily stable animal body plan.

## Results

### Identification of worm conserved non-coding elements

To identify highly conserved non-coding elements in the genome of *C. elegans*, we searched for sequences that contain large blocks of identity with the genome of *C. briggsae *and show no evidence of transcription. We used MegaBlast (with soft masking, e-value threshold of 0.001 and with the rest of the parameters set to the default values) to identify sequences that contain at least 30 (word seed size 30, W30) to 100 (word seed size 100, W100) consecutive nucleotides identical between the two nematode genomes, and removed any elements overlapping protein-coding exons, non-coding RNAs or repetitive sequences (see Materials and methods for details). We identified no non-coding elements with W100, 19 elements with W75, 304 elements with W50, 746 elements with W40 and 3,061 elements with W30. All further analysis was carried out on the W30 set. Of these elements, 69% are also found in the early draft genome sequence of *C. remanei*. We refer to these non-coding sequences conserved in all three genomes as worm CNEs (wCNEs), which comprise 1,460 intergenic elements with no evidence of transcription and 624 elements located in introns covering, in total, approximately 144 kb. These wCNEs have a mean length of 69 bp (minimum 30 bp, maximum 432 bp, median 59 bp) and a mean identity of 96% between *C. elegans *and *C. briggsae *with 990 elements being 100% identical between all three species. Using the PhastCons method [14], 93% of the total sequence contained in wCNEs is estimated to be under purifying selection rather than to be evolving neutrally. Moreover, this figure is probably an underestimation because the lack of sequence from other nematode species may result in an underestimation of branch lengths [14]. Therefore, the vast majority of wCNEs are likely to be functional elements under negative selection.

### wCNEs cluster around genes and are enriched on the X chromosome

**Figure 1:**
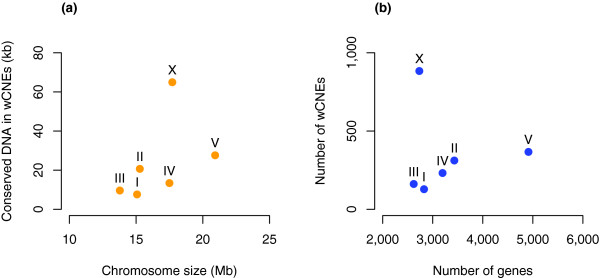
The distribution of CNEs in the *C. elegans *genome reveals enrichment on chromosome X. Chromosome X contains 884 out of 2,084 wCNEs. This enrichment for wCNEs on chromosome X cannot be explained by either **(a) **its size or **(b) **the number of genes it contains compared to the autosomes.

wCNEs are not distributed evenly along the chromosomes of *C. elegans*. Rather, they tend to reside in the gene-rich centers of the autosomes (Additional data files 1 and 2) and, as with human CNEs (hCNEs) [2-4], multiple wCNEs are often clustered around a single gene (mean of 1.7 and maximum of 14 wCNEs per gene). Moreover, 884 out of 2,084 wCNEs (42.4%) are found on the single *C. elegans *sex chromosome, which is more than expected by chance (*p *value < 0.001, based on 1,000 randomizations; Figure 1). The *C. elegans *sex chromosome is almost devoid of essential genes, and is instead enriched for genes with regulatory functions [20]. The enrichment of wCNEs on the X chromosome may, therefore, result from more of the genes on X requiring complex *cis*-regulatory architectures. This enrichment for CNEs on the X chromosome may also explain the larger synteny blocks that are observed on the X chromosome than on the autosomes in *C. elegans *[21]. In vertebrates it has been proposed that the requirement to maintain linkage between CNEs and their target genes places a constraint on chromosomal rearrangements [10] and this may also be occurring on the *C. elegans *X chromosome.

### Vertebrate and invertebrate CNEs share a striking nucleotide frequency pattern at their boundaries

**Figure 2:**
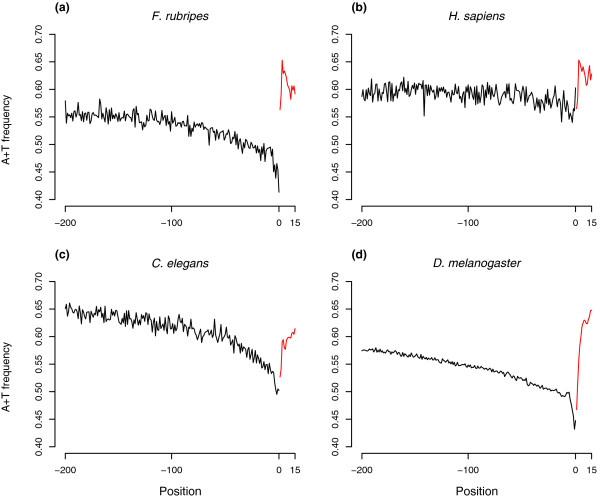
CNEs share a striking nucleotide signature from *C. elegans *to vertebrates. The plot shows the percentage of A+T nucleotides for 200 bp of sequence flanking CNEs (black) and 15 bp of CNE (red) at the CNE border defined by sequence conservation (the sequence on one end of each CNE is reverse complemented) for **(a) ***F. rubripes*, **(b) ***H. sapiens*, **(c) ***C. elegans *and **(d) ***D. melanogaster*. In all four species there is a decrease of A+T content in the 200 bp of sequence flanking the CNEs followed by a sharp A+T increase at the CNE border.

Vertebrate CNEs have a characteristic pattern of nucleotide composition, showing a sharp base composition change at their boundaries [22]. Fugu and human CNEs contain 59% and 62% A+T nucleotides [22], respectively, which is 6% and 3% above the genome averages [23,24]. A gradual G+C enrichment followed by a sharp AT-rich peak at the CNE boundaries marks the transition of base composition from the flanking DNA to the CNE DNA (Figure 2). The genome of *C. elegans *has increased A+T content (65%) compared to vertebrates. Yet wCNEs have an A+T content very similar to vertebrate CNEs (58%). Moreover, we find that worm CNEs also show a similar nucleotide frequency transition at their borders: there is a decrease of A+T content from the genome average (65%) down to 50% at the wCNE border followed by a sharp increase to 58% within the wCNE (Figure 2). Furthermore, the same signal is present at the boundaries of CNEs from *D. melanogaster *(Figure 2d) [25] (T Down, personal communication). The significance of this signal remains unknown, although its conservation from nematodes to humans indicates that it probably reflects a functional property of CNEs. For example, it might be a sign of a particular DNA conformation since AA/TT dinucleotides increase DNA rigidity, potentially making CNEs relatively rigid elements flanked by flexible DNA, or it may allow DNA unwinding and base unpairing [22]. The conservation of this signal from nematodes to humans could be useful for the discovery of functional non-coding elements less conserved than the CNEs (T Down, personal communication).

### wCNEs are associated with developmental transcription factors and signaling genes

CNEs in the human genome are associated with genes involved in the regulation of development and, in particular, with transcription factors ('trans-dev' genes) [2-4,6,7]. To assess whether CNEs are associated with certain types of genes in *C. elegans*, we spatially associated each wCNE to the protein-coding gene with the nearest transcription start site. The mean distance between a wCNE and the nearest transcription start site is 2,929 bp, with 1,206 (82.6%) of intergenic wCNEs lying more than 500 bp from the nearest transcription start site (Additional data file 3).

**Figure 3:**
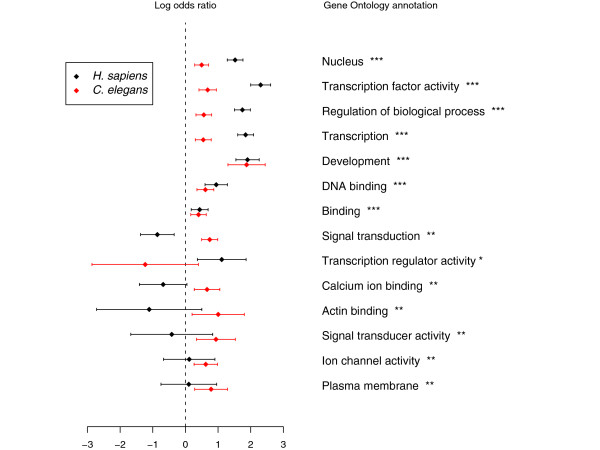
CNEs are associated with genes involved in transcription regulation and development in both *H. sapiens *and *C. elegans*. The log odds ratios and the 95% confidence intervals are shown for all GOslim terms that appear in the annotation of genes spatially associated with CNEs significantly more often than in the rest of the genome for *H. sapiens *(black) and *C. elegans *(red). GOslim terms marked with three asterisks are significantly enriched in both *H. sapiens *and *C. elegans *CNE genes; those marked with two asterisks are significantly enriched only in *C. elegans*; and the term with one asterisk is significantly enriched only in *H. sapiens*. The domains are ordered according to their *p *value in *H. sapiens *(lowest *p *value in *H. sapiens *at the top). All terms related to transcription factor activity and development (that is, 'trans-dev' genes [4]) show a strong bias in the annotation of genes near CNEs in both genomes. In the *C. elegans *gene set, there is also a trend for genes to be involved in signal transduction and ion binding. The GO terms shown in this figure constitute all GOslim terms (excluding the term 'biological-process') with a positive log odds ratio and *p *value ≤ 7.19 × 10-3 (5% false discovery rate cut-off) in either *H. sapiens *or *C. elegans*.

In both the human and the *C. elegans *genome the most significantly enriched functions, according to the Gene Ontology (GO) terms [26], for CNE-associated genes are related to transcription factor activity and development (Figure 3). For example, 2.82% (18/638) of genes associated with wCNEs are annotated with the GO term 'development', whilst only 0.63% (52/8,301) of all annotated genes in *C. elegans *are annotated with this term (*p *value = 6.13e-11 for log odds ratio = 1.87 and *p *value < 0.001, based on 1,000 randomizations). Similarly, 10.82% (69/638) of genes associated with wCNEs are annotated with the term 'transcription factor activity', whilst only 6.17% (512/8,301) of all annotated genes in *C. elegans *are annotated with this term (*p *value = 2.81e-7 for log odds ratio = 0.68 and *p *value = 0.006, based on 1,000 randomizations). The reverse association is also true: developmental genes in general are associated with wCNEs, as 34.62% (18/52) of annotated developmental genes (that is, annotated with the GO term 'development') are associated with wCNEs while only 7.69% (638/8,301) of all annotated genes in *C. elegans *are associated with wCNEs. Glazov *et al*. [13] have noted a similar trend for elements conserved between two very closely related *Drosophila *species, indicating that the association of highly conserved non-coding elements with trans-dev genes is a property conserved from worms to humans.

In addition, wCNE-associated genes are enriched for cell-signaling GO terms, which has also been noted for the elements in *Drosophila *[13], but is less striking in humans[13]. Nonetheless, several examples of major signaling genes involved in development are associated with CNEs in the human genome, with a classic example being the sonic hedgehog gene at 7q36.3 [10]. This difference is an intriguing result considering that the human genome contains more signaling genes (1,790/15,023 = 11.92% of human genes annotated with the term 'signal transduction') than the *C. elegans *genome (599/8,301 = 7.22%), whereas there are fewer signaling genes among the human CNE-associated genes (15/274 = 5.47%) than the wCNE-associated genes in *C. elegans *(84/638 = 13.17%). A possible explanation for this difference is that signaling genes in vertebrates are associated with elements less conserved than the CNEs we previously identified. In support of this hypothesis, a set of vertebrate non-coding elements identified with less stringent criteria are significantly enriched for the GO term 'signal transduction' [27].

To further analyze the types of genes associated with CNEs, we looked at the InterPro protein domains [28] encoded by these genes. Both *Homo sapiens *and *C. elegans *CNEs are enriched in the neighborhoods of genes encoding DNA-binding transcription factor domains (including Homeodomain-like, Winged-helix repressor DNA-binding, Zinc finger C2H2-type and HMG1/2; Additional data file 4). We also examined the enrichment for transcription factors among the wCNE-associated genes using predicted transcription factors from two high-quality databases: DBD, a database of computationally predicted transcription factors through homology to known DNA-binding domains [29]; and wTF2.0, a compendium of computationally and manually curated transcription factors in *C. elegans *[30]. Out of 1,241 of the wCNE-associated genes, 108 (8.7%) are annotated as transcription factors according to DBD and 137 out of 1,241 (11.0%) of the wCNE-associated genes are annotated as transcription factors according to wTF2.0, both being significantly higher than the proportion in the genome (Additional data file 5).

Both *H. sapiens *and *C. elegans *CNEs are also associated with genes encoding cell-signaling domains, although, as also noted from the GO terms, this is more pronounced in *C. elegans*. These domains include those found in extracellular proteins, cell surface receptors, and intracellular signaling proteins. The lack of thoroughly annotated sets of signaling genes could potentially exaggerate differences between human and worm CNE-associated genes.

Human CNEs are not always found directly adjacent to their most likely target genes [6,7]. It is also possible that CNEs may regulate more than one gene, for example, in the case of bidirectional promoters. Therefore, these statistics probably underestimate the true association of CNEs with developmental regulatory genes. We conclude that CNEs are associated with genes involved in transcription regulation and development and, to a certain degree, cell-signaling in both vertebrates and invertebrates, although the association with cell signaling genes appears to be stronger in invertebrates.

### Vertebrate and invertebrate CNEs target a common set of core developmental genes

Most strikingly, we find that many of the genes associated with CNEs in the *C. elegans *genome are the direct orthologs of CNE-associated genes in the human genome. Of 397 human CNE-associated genes, 190 have identifiable orthologs in *C. elegans *and, of these, 60 are also associated with wCNEs in *C. elegans*. This is much greater than expected by chance (*p *< 0.001, by randomization). For example, the *C. elegans *gene *mab-18 *is associated with ten wCNEs and its human ortholog PAX6 is associated with two hCNEs. For worm CNE-associated genes that have been duplicated in the vertebrate lineage, multiple paralogs are often associated with hCNEs. For example, the *C. elegans *gene *sem-4 *is associated with four wCNEs, and has four human orthologs. Of these, SALL1 is associated with two hCNEs, SALL3 with eleven hCNEs and SALL4 with one hCNE.

Remarkably, of the 60 human CNE-associated genes that have *C. elegans *orthologs that are associated with wCNEs, 40 also have orthologs in *Drosophila *that are associated with the conserved elements identified by Glazov *et al*. [13]. In summary, 40 of 156 human CNE-associated genes that have orthologs in both *C. elegans *and *D. melanogaster*, are also associated with CNEs in these two species. These genes represent a core set of developmental regulatory genes that are associated with CNEs across three different animal phyla (Table 1). Thus, despite the extensive evolutionary distance and duplication events that have occurred since the divergence of *C. elegans*, *D. melanogaster *and *H. sapiens*, a core set of orthologous genes are associated with highly conserved non-coding elements in all three organisms.

### wCNEs identify transcriptional enhancer sequences and may function as transcription factor binding sites

**Figure 4:**
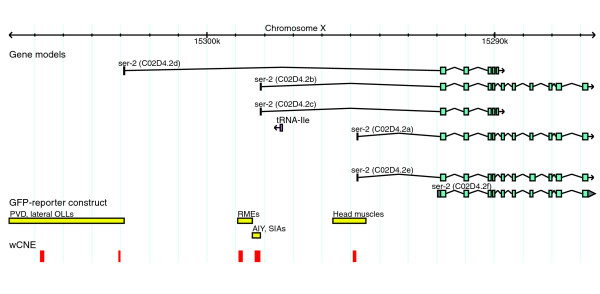
CNEs identify previously characterized enhancer sequences and when located in introns are associated with alternative transcriptional start sites. Five wCNEs are contained within four elements that regulate ser-2, the *C. elegans *ortholog of human serotonin receptor 1A. The products of ser-2 were identified as components of the AIY interneuron gene battery in *C. elegans *[60]. ser-2 has at least three alternative transcription start sites that produce a number of different gene products, considered to be expressed in different but overlapping regions [61]. Remarkably, each of the alternative transcription start sites is marked by a wCNE in the proximal upstream region, with additional wCNEs lying further away, highlighting the underlying *cis*-regulatory elements. The upstream sequences of each of the alternative transcription start sites were defined by deletion analysis [61]. One of the wCNEs lies within an approximately 280 bp element driving expression in the AIY and SIA neuronal cellular subtypes. A second wCNE lies within an approximately 520 bp element driving expression in the RME neurons and also, consistently, in other unidentified neurons. A third wCNE lies within an approximately 1,150 bp element driving expression in the head muscles. Two more wCNEs are contained within a region driving expression in PVD and lateral OLL neurons. Only the experimentally tested constructs that overlap wCNEs are shown in this diagram.

Human CNEs have been proposed to act as *cis*-elements that regulate the transcription of developmental genes, and of the relatively few vertebrate CNEs that have been tested, the majority can act as tissue-specific enhancers when co-injected with a reporter gene in zebrafish or in transgenic mice [4,7-10]. Therefore, we reasoned that, if worm CNEs also function as enhancers, then they should overlap multiple previously characterized enhancer sequences in the worm genome. By using literature searches, we compiled a list of 17 *C. elegans *genes with extensively dissected *cis*-regulatory sequences. We found that six of these genes are associated with wCNEs, and that, in five of these six cases, the wCNEs are contained within the defined enhancer regions (Additional data file 6). For example, the gene *ser-2 *is associated with five wCNEs, and each of these wCNEs lies within a genomic region that acts as a transcriptional enhancer for a different tissue or cell type (Figure 4). This provides good evidence that CNEs can act as transcriptional enhancers *in vivo*.

**Figure 5:**
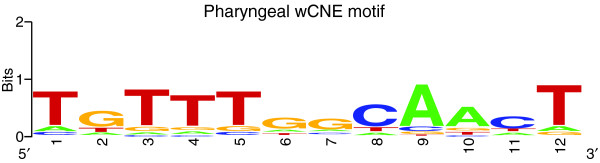
Worm CNEs are enriched for transcription factor binding sites. Sequence logo representation of the motif significantly overrepresented in wCNEs associated with pharyngeal genes, according to the Weeder motif discovery algorithm [32]. The first six bases of this motif (with consensus TGTTTGGCAACT) agree with the first six bases of the consensus binding site of the PHA-4 transcription factor (TRTTKRY, where R = A/G, K = T/G, and Y = T/C) [33, 34]. Note that the seventh position of the predicted motif has low information content, indicating that sites with differences in this position are still likely to represent variants of the same transcription factor binding site.

The simplest hypothesis for how CNEs function is that they encode arrays of transcription factor binding sites. If this were the case, then CNEs associated with genes known to be expressed in a particular tissue type should be enriched for DNA binding sites for transcription factors regulating the co-expression of these genes in that tissue. To test this hypothesis, we used DNA microarray data [31] to identify 54 wCNE-associated genes that are expressed in the *C. elegans *pharynx. These genes are associated with a total of 120 wCNEs (from here on referred to as 'pharyngeal wCNEs'). Our set of pharyngeal wCNEs contains 40 intronic and 80 intergenic wCNEs, ranging in size from 31 bp to 216 bp (mean = 68.2 bp; median = 60 bp). It is important to note that many of the intergenic wCNEs in this set lie further than the classical 'promoter region' (often described as the first 500 bp to 1,000 bp upstream of a gene), with pharyngeal wCNEs ranging from 27 bp to 9,577 bp (mean = 2,970 bp; median = 2,053 bp) from the associated pharyngeal gene. To identify putative transcription factor binding sites in the pharyngeal wCNEs, we searched for overrepresented sequence motifs using the Weeder motif discovery algorithm, which searches for overrepresented motifs and then carries out a post-processing step to identify similar ('redundant') motifs among the highest scoring motifs [32]. Weeder identified a single redundant motif that is significantly enriched in these sequences (*p *< 0.002). Strikingly, this motif (Figure 5) is very similar to the consensus binding site of the pharyngeal transcription factor PHA-4. PHA-4 is the major specifier of pharyngeal cell identity in *C. elegans *[33,34], suggesting that occurrences of this motif in wCNEs represent genuine PHA-4 binding sites. Indeed, inspection of the seven highest scoring occurrences of the motif in the pharyngeal CNEs (Table 2) revealed that one of the predicted sites lies 1.2 kb upstream of the gene *ceh-22*, within a 30 bp pharyngeal muscle enhancer previously shown to be bound by PHA-4 [35] (annotated in WormBase as two overlapping PHA-4 binding sites with WormBase identifiers WBsf019089 and WBsf019090). Therefore, by searching for overrepresented motifs in a set of wCNEs associated with genes expressed in the pharynx, we were able to identify the binding site for the transcription factor that acts as the major specifier of pharyngeal cell identity. Taken together with the identification of other wCNEs within previously characterized enhancers, this suggests that wCNEs represent enhancer sequences that function (at least partially) by encoding transcription factor binding sites.

### Intronic wCNEs likely function as enhancers for downstream alternative transcription start sites

Almost a third of wCNEs (624/2,084) are located in introns. To investigate whether intronic wCNEs represent a separate type of element, we examined whether they are associated with particular classes of transcripts. We found that there is a strong association between the presence of an intronic wCNE and genes that are known to produce multiple different transcripts (57% of genes containing intronic wCNEs have documented alternative transcripts, compared to 19% of all multi-exon genes). Moreover, in 70% of the cases of alternatively spliced genes containing intronic wCNEs, the gene encodes an alternative first exon (compared to 35% of all genes with alternative transcripts). This suggests that intronic wCNEs are strongly associated with genes with alternative first exons and, therefore, that intronic wCNEs may act as enhancers for downstream alternative start sites. In support of this hypothesis, in 78% of cases the intronic wCNE is located upstream of the alternative first exon (see Figure 4 for examples). Therefore, we do not believe that, in general, intronic wCNEs regulate alternative splicing. Rather, we suggest that, at least in *C. elegans*, the majority of intronic wCNEs, like intergenic wCNEs, probably function as *cis*-regulatory transcriptional enhancers, but for downstream alternative transcriptional start sites.

## Discussion

### Shared properties of nematode and vertebrate CNEs

We have identified a set of highly conserved non-coding sequences (wCNEs) in the genome of *C. elegans*. Just as with CNEs in the human genome, these wCNEs are clustered around genes that encode regulators of development, especially transcription factors and signaling genes. Both human and worm CNEs share striking nucleotide frequency patterns at their boundaries and are similarly AT-rich, despite differences in the background A+T content of their genomes. Worm CNEs overlap many independently identified *cis-*regulatory elements, and vertebrate CNEs can act as tissue-specific enhancers in transient zebrafish assays. It seems likely, therefore, that human and worm CNEs function analogously as *cis*-elements that regulate the transcription of a core set of developmental regulatory genes. Consistent with this model, intronic wCNEs are very strongly associated with downstream alternative transcriptional start sites, suggesting that they too probably function as tissue-specific *cis*-regulatory elements.

How do CNEs regulate gene-expression? The simplest model is that CNEs encode transcription factor binding sites. In support of this model, we find that wCNEs associated with genes expressed in the pharynx are significantly enriched for a DNA motif that matches the binding-site of the major pharynx specifying transcription factor PHA-4. However, it is still difficult to reconcile the length and level of sequence conservation of CNEs with the known sequence constraints of transcription factor binding sites, especially since conservation of individual transcription factor binding sites has been found unnecessary for conservation of enhancer function (for example, [36]). Therefore, CNEs may represent very dense, potentially overlapping transcription factor binding sites. If this scenario were true, differences in the number of overlapping constraints between different *cis*-elements would manifest as differences in the degree of sequence conservation of these elements, with CNEs representing the most extreme cases. Indeed, reducing the stringency of the conservation threshold (either by relaxing the similarity search criteria [4,27] or by comparing less divergent species [37]) often reveals additional or longer non-coding elements. Alternatively, CNEs may also encode some additional regulatory function. For example, it is possible to envisage mechanisms involving sequence recognition between homologous chromosomes (for example, 'transvection' [38]) that would require sequence identity to be maintained between the maternal and paternal genomes.

Remarkably, of the 190 genes that are associated with CNEs in humans and have orthologs in *C. elegans*, 60 have orthologs that are also associated with CNEs in *C. elegans*. This suggests that unrelated CNEs may be associated with a core set of regulatory genes in many divergent animal species. In support of this, 40 of these 60 genes are also orthologous to CNE-associated genes in *D. melanogaster*. Such an overlap between the sets of CNE-associated genes from three animal phyla is very unlikely to have arisen by chance, and suggests that a core set of developmental regulatory genes may be associated with CNEs across all animal lineages.

Because of its genetic tractability and reduced intergenic distances, we propose that *C. elegans *will serve as an excellent model organism for further understanding the mechanism by which CNEs regulate gene expression. The dissection of CNEs in parallel in different animals using both computational and experimental approaches would provide us with valuable insight into the evolution of the regulatory networks that control the development of the metazoan body plan.

Since many of the genes associated with CNEs encode for transcription factors that control early development, it is possible that CNEs themselves are bound by these transcription factors. Orthologous transcription factors are not only present in most metazoan lineages, but also often have highly conserved DNA-binding domains (for example, the DNA-binding domains of orthologous HOX [39], FOXA [33,34] and Brachyury T-box [40] proteins). It is tempting, therefore, to speculate that CNEs might function as enhancers even when tested in different animal lineages. A small number of reporter gene assays testing enhancers of regulatory genes from vertebrates in flies have shown positive results (for example, [41-43]), indicating that the regulators of these vertebrate enhancers are also present in flies. However, we would not expect alternative CNEs from different animals to drive the same expression patterns, reflecting differences in the body plans of different animal lineages.

### CNEs and the evolution of animal body plans

The evolution of *cis*-regulatory elements is an important driving force in the evolution of gene regulatory networks (GRNs). In the case of multicellular animals, the initial assembly and subsequent modifications of *cis*-elements for key developmental control genes probably allowed the 're-wiring' of developmental GRNs and, hence, the evolution of new animal body plans [44]. In this way, regulatory genes became associated with alternative sets of *cis*-elements in different animal lineages and these *cis*-elements now define the core GRNs of each animal body plan. We propose that CNEs represent the 'hard-wired' sequence traces of these core animal group-specific GRNs. The alternative core GRNs of different animal lineages are reflected in their having alternative CNEs. However, because of their co-evolution from a common metazoan ancestor, the core GRNs of different animal groups often utilize the same regulatory genes. As a result, distinct yet parallel sets of CNEs have become irreversibly associated with the same genes that coordinate core developmental networks in diverse animal groups. Indeed, this evolution of regulatory elements may underlie the astounding diversification of animal body plans that was seen during the Cambrian period approximately 550 million years ago.

## Materials and methods

### Identification of conserved non-coding elements in *C. elegans*

DNA sequences and annotation files for the *C. elegans *genome (release WS140), the DNA sequence for the *C. briggsae *genome (release cb25) and the repeat-masked sequence of the *C. remanei *genome (downloaded on 30 October 2005) were retrieved from WormBase [45]. The sequence of each *C. elegans *chromosome was split into 500 kb fragments overlapping by 200 bp. We searched for local similarity between each 500 kb sequence fragment from *C. elegans *against the genome of *C*. briggsae using MegaBlast (version 2.2.6) [46]. We performed MegaBlast searches with soft masking, e-value threshold of 0.001 and word seed size 100 bp (W100), 75 bp (W75), 50 bp (W50), 40 bp (W40) and 30 bp (W30). Where overlapping regions of the query (*C. elegans*) sequence matched more than one location in the *C. briggsae *genome, these regions of the query were merged, resulting in non-overlapping elements. Conserved elements were annotated according to the set of WormBase features provided in Additional data file 7. Elements not overlapping any of these features were marked as 'unannotated' elements and elements within introns of protein-coding genes were only annotated as 'intronic' if they did not overlap any type of exons or repeats (Additional data file 7). Our definition of unannotated and intronic conserved elements is very conservative, so that any amount of overlap between a conserved element and a genomic feature, such as any type of exon, a match to an expressed sequence, a predicted gene, or a repeat is considered sufficient to mask this element as exonic or repetitive. Unannotated and intronic elements were further scanned for missed repeats using RepeatMasker (version 3.0.8, slow/sensitive option, using Crossmatch) [47] using both the *C. elegans *repeat library distributed with the program and the *C. briggsae *library downloaded from WormBase [18]. The remaining elements were scanned for missed tRNAs using tRNAscan-SE (v.1.11) [48]. In addition, 36 elements with a BLAST match in Rfam [49], the microRNA registry database [50] and EMBL expressed sequence tags (downloaded on 21 April 2005) were removed (e-value threshold of 0.0001). We then checked whether the remaining unannotated or intronic conserved elements found in *C. elegans *and *C. briggsae *were similarly conserved in the genome of *C. remanei *using MegaBlast, with soft masking, word seed length of 30 bp and e-value threshold 0.0001. Of the elements conserved between *C. elegans *and *C. briggsae*, 69% were also found in *C. remanei *using the same similarity search criteria. The sequences of the final set of 2,084 wCNEs are provided in Additional data file 8. Finally, we searched the set of 2,084 wCNEs for sequence similarity against the human CNEs (1,373 sequences from Woolfe *et al*. [4]) using BlastN (version 2.2.6) [51] and found no significant hits (e-value threshold = 0.0001).

We compared the final set of elements conserved between all three *Caenorhabditis *species with elements identified as conserved by WABA, a sensitive alignment method designed to find homologous regions between the *C. elegans *and the *C. briggsae *genome and annotate them as 'strongly conserved', 'weakly conserved' or 'coding' using information on conservation and the third base 'wobble' of protein-coding sequences [12]. Of all base pairs in wCNEs, 97% are contained within alignments classified as strongly conserved, 0.6% are within alignments classified as coding and 6% are within alignments classified as weakly conserved.

We also calculated the overlap between wCNEs and elements predicted as conserved using PhastCons. PhastCons [14] is a statistical method that scores sequences in alignments according to how much more likely it is that they are conserved than that they are evolving neutrally, based on a phylogenetic hidden Markov model. Elements predicted to be conserved by PhastCons based on *C. elegans*-*C. briggsae *BLASTZ alignments [52] were retrieved through the UCSC Genome Browser (table PhastConsElements).

### Genomic distribution and sequence analysis of wCNEs

The clustering of wCNEs along the *C. elegans *chromosome and the comparison of the distances between CNEs in the human and the *C. elegans *genome were calculated as described in Woolfe *et al*. [4]. We assessed the enrichment of wCNEs on chromosome X using a randomization test. We generated 1,000 sets of 2,084 (that is, the same number as the wCNEs) random locations in the *C. elegans *genome, making sure that the random locations lie within non-coding and non-repetitive regions. The random sets had, on average, 487.3 wCNEs on X (minimum = 432; maximum = 550). Therefore, the enrichment of wCNEs on chromosome X is highly significant (*p *value < 0.001).

The A+T nucleotide content in the 200 bp flanking CNEs and the first 15 bp of CNEs were calculated according to Walter *et al*. [22]. In brief, 215 bp of sequence from one CNE end and 215 bp of reverse complemented sequence from the other CNE end were aligned according to the first position of each CNE. The percentage of A+T nucleotide composition was calculated and plotted for each position along this 215 bp alignment.

### Analysis of CNE-associated genes in *C. elegans*, *D. melanogaster *and *H. sapiens*

For each of the 2,084 wCNEs, we identified the protein-coding genes with the nearest transcription start site (TSS) according to the WormBase annotation (WS140; Additional data file 9). We assigned 1,241 genes to the wCNEs. As fly CNEs we used the 20,301 intergenic and intronic 'ultraconserved' (uc) elements between *D. melanogaster *and *D. pseudoobscura *with size ≥50 bp from Glazov *et al*. [13]. We associated each uc-element to the gene with the nearest transcription start site (according to the fly genome annotation release dm1). We assigned 3,750 genes to fly uc-elements. Similarly, we identified the nearest protein-coding genes to the 1,373 human elements conserved between human and Fugu from Woolfe *et al*. [4] according to human genome NCBI35 accessed via Ensembl [53] v35 (397 genes) (Additional data file 10).

The GO annotation files for *C. elegans *(revision 1.55) and human (revision 1.22) were downloaded directly from the GO website [26]. Only GO terms inferred automatically (GO evidence code IEA) were used in our analysis because of the heavy bias of RNAi phenotypes on the GO annotations of the genes in *C. elegans *(our unpublished observation). To increase the signal, GO terms were converted to the higher-level GOslim terms and these are the terms we have used in this paper. GOslim term associations and counts for *C. elegans *and human genes were calculated using the Perl script map2slim from the go-perl package and the generic GOslim (revision 1.116) [54]. Out of 397 human genes and 1,241 *C. elegans *genes, 274 and 638, respectively, were assigned a GOslim term. We retrieved protein domains for the *C. elegans *and the human genes from Ensembl. Out of 397 human genes and 1,241 *C. elegans *genes spatially associated with CNEs, 316 and 877, respectively, were annotated with at least one InterPro domain. To increase the signal, each domain was converted to the top-level parent domain according to the InterPro protein domain annotation using a custom Perl script. The following analysis was carried out for all top-level InterPro domains found in at least ten genes in the human and the *C. elegans *genomes. For each type of annotation (i.e. each GOsilm term and each InterPro parent domain), we calculated the log odds ratio log((a × b)/(c × d)) , where   a is the number of genes in the CNE-associated gene set with the specific annotation, b is the number of annotated genes without the specific annotation and not in the CNE-associated genes set, c is the number of   genes with the specific annotation but not in the CNE-associated gene set, and d is the number of remaining annotated genes without the specific annotation in the CNE-associated gene set. The log odds ratios, confidence   intercals (CI) and p-values were calculated using the R statistical package [55] (according to a two-tailed test). The *p *value threshold at the 5% false discovery rate (FDR) cut-off was calculated according to the false discovery rate method by Benjamini and Hochberg [56]. We also carried out a randomization test to check how likely it is to get by chance the same proportion of genes annotated with the GO terms 'transcription factor activity' and 'development' as we did for the wCNE set. To do this, we generated 1,000 sets of 2,084 (that is, the same number as the wCNEs) random locations in the *C. elegans *genome, making sure that the random locations lie within non-coding and non-repetitive regions. For each random location, we then retrieved the gene with the nearest transcription start site. Finally, for each set, we counted the proportion of genes annotated with the GO terms 'transcription factor activity' and 'development'.

Orthologous gene clusters were retrieved from Inparanoid (version 4.0) [57]. The Inparanoid dataset contains clusters of orthologous proteins between pairs of genomes. There are 4,558 Inparanoid clusters of orthologous proteins from *C. elegans *and human that contain 8,846 human proteins and 5,614 *C. elegans *proteins. Of the human protein-coding genes with *C. elegans *orthologs and the *C. elegans *protein-coding genes with human orthologs, 190 and 424, respectively, are associated with CNEs. For *D. melanogaster *and *H. sapiens*, there are 5,497 Inparanoid clusters of orthologs that contain 8,960 human proteins and 6,170 *D. melanogaster *proteins. Of the human protein-coding genes with *D. melanogaster *orthologs and the *D. melanogaster *protein-coding genes with human orthologs, 215 and 1,254, respectively, are associated with CNEs/uc-elements. To evaluate the significance of the overlap between CNE-associated genes in human and *C. elegans*, we performed 1,000 randomizations, randomly picking 424 *C. elegans *genes from those in the Inparanoid clusters and counting how many of them have an ortholog among the 190 human CNE-associated genes. The same overlap of CNE-associated genes in the two genomes was never seen in 1,000 randomizations. Similarly, we performed 1,000 randomizations, randomly picking 1,254 *D. melanogaster *genes and counting how many of them have an ortholog among the 215 human CNE-associated genes. Again, the same overlap of CNE-associated genes in the two genomes was never seen in 1,000 randomizations.

### Motif discovery in pharyngeal gene-associated CNEs

Genes expressed in the pharynx were identified by microarray analysis in [31]. The 120 wCNEs associated with pharyngeal genes were submitted to a local installation of Weeder (version 1.3) [32]. We performed an 'extra' mode search (that is, looking for motifs 6 bp long with 1 mismatch, 8 bp long with 3 mismatches, 10 bp long with 4 mismatches and 12 bp long with 4 mismatches), looking for motifs in both strands and reporting back 50 motifs. Post-processing of the identified motifs carried out by Weeder returned one 'redundant' motif in the form of a position weight matrix (PWM) and seven high scoring matches of this PWM in the input set of sequences. The significance of the motif identified by Weeder was estimated using the Weeder *p *value calculator [58]. The high scoring matches of the PWM in the pharyngeal wCNEs are shown in Table 2. The sequence logo for this PWM was created using WebLogo [59].

## Additional data files

The following additional data are available with the online version of this paper. Additional data file 1 is a figure showing the distribution of wCNEs along each chromosome. Additional data file 2 is a figure showing the distribution of distances between CNEs in both the *C. elegans *and the *H. sapiens *genomes and for randomized CNE locations. Additional data file 3 is a figure showing the distribution of distances between intergenic wCNEs and their nearest genes. Additional data file 4 is a table showing all the top-level InterPro protein domains significantly enriched in CNE-associated genes compared to the rest of the genes in the **(a) ***H. sapiens *and **(b) ***C. elegans *genomes. Additional data file 5 is a table showing the enrichment for transcription factors among wCNE-associated genes, using two different collections of predicted transcription factors from the *C. elegans *genome. Additional data file 6 is a table showing wCNEs overlapping known *cis*-regulatory elements. Additional data file 7 is a table showing the annotation features from WormBase that were used to annotate wCNEs. Additional data file 8 contains the sequences of the 2,084 wCNEs. Additional data file 9 is a table with the coordinates of the wCNEs in the *C. elegans *genome, their nearest genes and their human orthologs. Additional data file 10 is a table with the coordinates of the human CNEs from Woolfe *et al*. [4] with their nearest genes assigned using the same method as for the wCNEs.

## Supplementary Material

Additional data file 1 Distribution of wCNEs along each chromosome. Click here for file

Additional data file 2 Distribution of distances between CNEs in both the *C. elegans *and the *H. sapiens *genomes and for randomized CNE locations. Click here for file

Additional data file 3 Distribution of distances between intergenic wCNEs and their nearest genes. Click here for file

Additional data file 4 Top-level InterPro protein domains significantly enriched in CNE-associated genes compared to the rest of the genes in the (a) *H. sapiens *and (b) *C. elegans *genomes. Click here for file

Additional data file 5 Enrichment for transcription factors among wCNE-associated genes, using two different collections of predicted transcription factors from the *C. elegans *genome. Click here for file

Additional data file 6 wCNEs overlapping known *cis*-regulatory elements. Click here for file

Additional data file 7 Annotation features from WormBase that were used to annotate wCNEs. Click here for file

Additional data file 8 Sequences of the 2,084 wCNEs. Click here for file

Additional data file 9 Coordinates of the wCNEs in the *C. elegans *genome, their nearest genes and their human orthologs. Click here for file

Additional data file 10 Coordinates of the human CNEs from Woolfe *et al*. [4] with their nearest genes assigned using the same method as for the wCNEs. Click here for file

## Figures and Tables

**Table 1:** Orthologous genes associated with CNEs (and uc-elements) in humans, flies and worms

	*C. elegans*		*D. melanogaster*		*H. sapiens*	
Cluster number	Gene name	Number of associated wCNEs	Gene symbol	Number of associated uc-elements	Gene name	Number of associated hCNEs

1	ZC123.3	4	*zfh2*	2	*ATBF1*	3
					*ZFHX4*	10
2	*ceh-31*	3	*B-H1*	40	*BARHL2*	18
	*ceh-30*	2	*B-H2*	39		
3	*ceh-44*	1	*ct*	38	*CUTL2*	2
4	*unc-3*	5	*kn*	1	*EBF3*	15
5	C18B12.3	1	*al*	17	ENSG00000165606	5
6	*egl-43*	1	CG31753	9	*EVI1*	2
					*PRDM16*	1
7	*lin-39*	1	Scr	21	*HOXA5*	2
					*HOXB5*	2
					*HOXC5*	2
8	*irx-1*	1	*caup*	12	*IRX4*	8
			*mirr*	23	*IRX6*	3
			*ara*	5		
9	*mab-21*	2	CG4766	6	*MAB21L1*	5
			*mab-2*	9	*MAB21L2*	4
10	*cog-1*	3	*HGTX*	20	*NKX6-1*	7
11	*nhr-67*	1	*dsf*	3	*NR2E1*	1
12	*nhr-6*	2	*Hr38*	8	*NR4A2*	12
13	*vab-3*	10	*toy*	1	*PAX6*	2
14	*unc-30*	1	*ptx1*	11	*PITX2*	3
15	*unc-86*	2	*acj6*	6	*POU4F1*	1
					*POU4F2*	1
16	*ptc-1*	1	*ptc*	4	*PTCH*	3
17	*egl-27*	3	*gug*	7	*RERE*	1
18	*unc-10*	1	*Rim*	3	*RIMS2*	1
19	*rnt-1*	3	*run*	13	*RUNX3*	1
20	*sem-4*	4	*Salm*	32	*SALL1*	5
					*SALL3*	11
					*SALL4*	1
21	*sox-3*	2	*SoxN*	13	*SOX1*	2
					*SOX2*	2
22	*tbx-2*	4	*bi*	28	*TBX2*	1
23	K06A1.1	1	*AP-2*	5	*TFAP2A*	2
					*TFAP2D*	2
24	*zag-1*	4	*zfh1*	7	ZFHX1B	18
25	*ref-2*	1	*opa*	11	*ZIC1*	1
					*ZIC2*	1
					*ZIC4*	4
26	*tlp-1*	1	*elB*	9	*ZNF503*	3
			*noc*	35	*ZNF703*	4

**Table 2:** Occurrences of a sequence motif overrepresented in wCNEs associated with pharyngeal genes

wCNE coordinates	Strand	Matching sequence	Position	Score	wCNE distance from TSS	Gene name
IV:3776258..3776298	+	TATTTAGCATCT	9	85.59	9,435	*vab-2*
IV:8369551..8369581	-	TTTTTTGCAACT	3	91.65	347	D2096.6
V:10673732..10673841	-	TGTTTGTCCACT	15	87.26	1,202	ceh-22*
V:13217316..13217419	+	TGTTTGGCAACT	23	100	3,588	F57B1.6
X:2215856..2215898	-	TGTTTTGAAATT	12	85.67	230	*peb-1*
X:6621897..6621968	-	TTTATGGCAACT	47	88.99	826	C25B8.4
X:7457940..7457992	+	TGTTTGACAATT	5	91.56	2,212	*sox-2*

We used the Weeder motif discovery program to search for overrepresented motifs in all wCNEs spatially associated with genes predicted to be expressed in the pharynx based on microarray data [31]. From this dataset, Weeder identified a motif very similar to the consensus binding site for PHA-4, the master specifier of pharyngeal cell identity (TRTTKRY, where R = A/G, K = T/G, and Y = T/C) [33, 34]. This table shows the coordinates (WormBase version WS140) of the wCNEs that contain matches to the overrepresented motif, the coordinates of the matches within the wCNEs, the Weeder scores of the matches to the motif, the distances (in bp) between the wCNEs and the transcription start site (TSS) of the associated genes and the names of the associated genes. The predicted site in the element 1.2 kb upstream of *ceh-22 *(marked with an asterisk) lies within a 30 bp pharyngeal muscle enhancer bound by PHA-4 [35].
