# Supplementary material for: Parallel evolution of conserved non-coding elements that target a common set of developmental regulatory genes from worms to humans
Source: Genome Biol. 2007 Feb 2;8(2):R15. doi: 10.1186/gb-2007-8-2-r15 (PMC1852409; doi:10.1186/gb-2007-8-2-r15)

Figure S1. The distribution of wCNEs along each chromosome reveals clustering in the centres of autosomes. The cumulative wCNE occurrence (y-axis) is shown along each of the *C. elegans* chromosomes (x-axis).

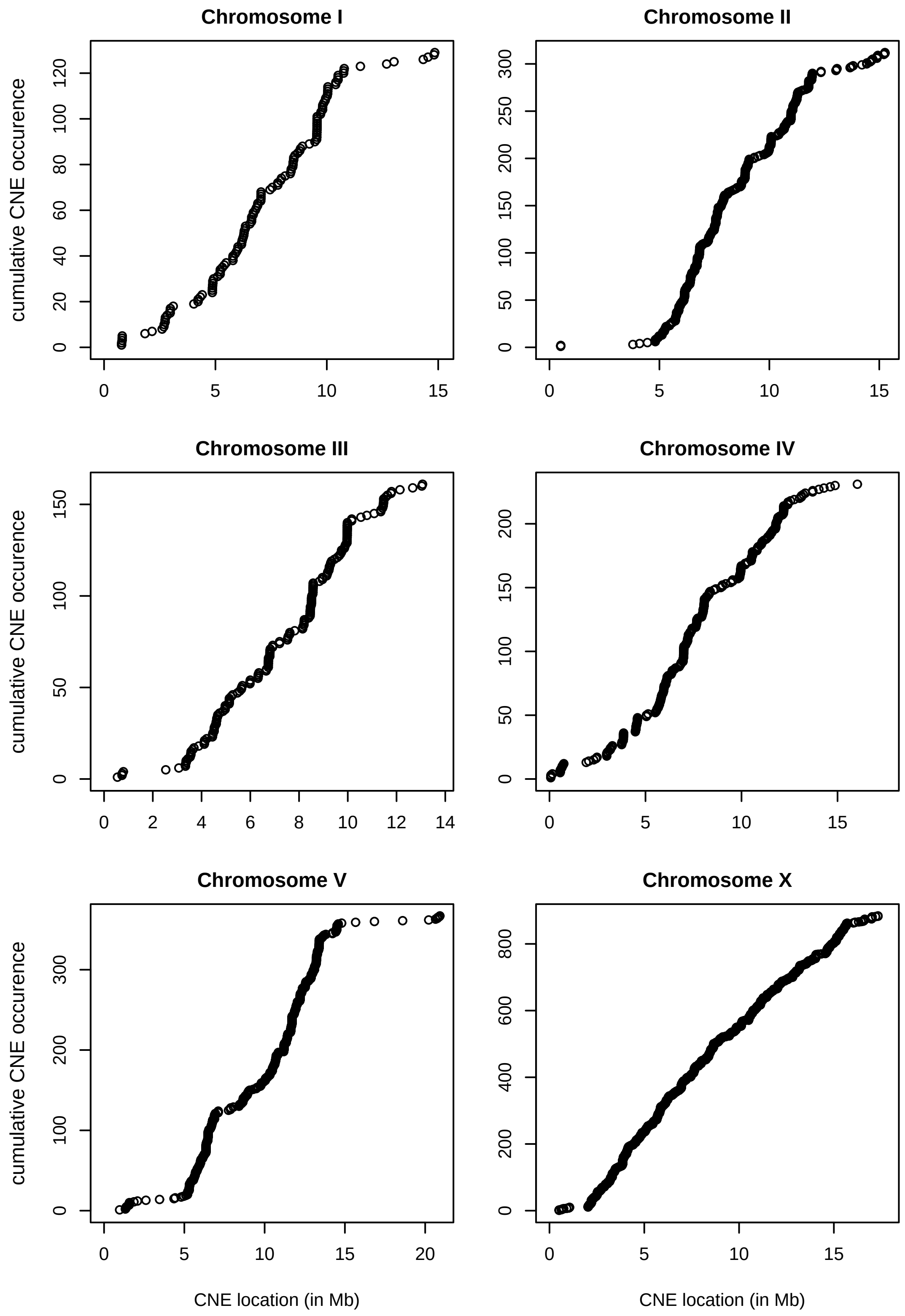

Supplement: Additional data file 1 — Distribution of wCNEs along each chromosome. [file gb-2007-8-2-r15-S1.pdf]
