# Supplementary material for: Parallel evolution of conserved non-coding elements that target a common set of developmental regulatory genes from worms to humans
Source: Genome Biol. 2007 Feb 2;8(2):R15. doi: 10.1186/gb-2007-8-2-r15 (PMC1852409; doi:10.1186/gb-2007-8-2-r15)

Figure S2. The distribution of distances between CNEs reveals clustering of CNEs in both the *C. elegans* and the *H. sapiens* genome. The median distance between consecutive CNEs is 8 kb for *C. elegans* and 49 kb for *H. sapiens*. We tested whether CNEs are randomly distributed according to Woolfe *et al*, 2005. In brief, a new random location was allocated uniformly for each CNE within its chromosome. This process was repeated 1,000 times for each chromosome. Consecutive CNEs that were closer to each other than the median inter-CNE distance were considered part of the same cluster and mean cluster sizes were calculated for the randomized CNE locations. The distributions of cluster sizes for real CNE locations in *C. elegans* (top left) and *H. sapiens* (top right) were compared against the distributions of cluster sizes for randomized CNE locations in *C. elegans* (bottom left) and *H. sapiens* (bottom right), respectively, using the chi-squared test. The comparison showed that in both the *C. elegans* and the *H. sapiens* genome CNEs are more clustered than expected by chance (p = 7.7e-20 and p = 2.6e-16, respectively).

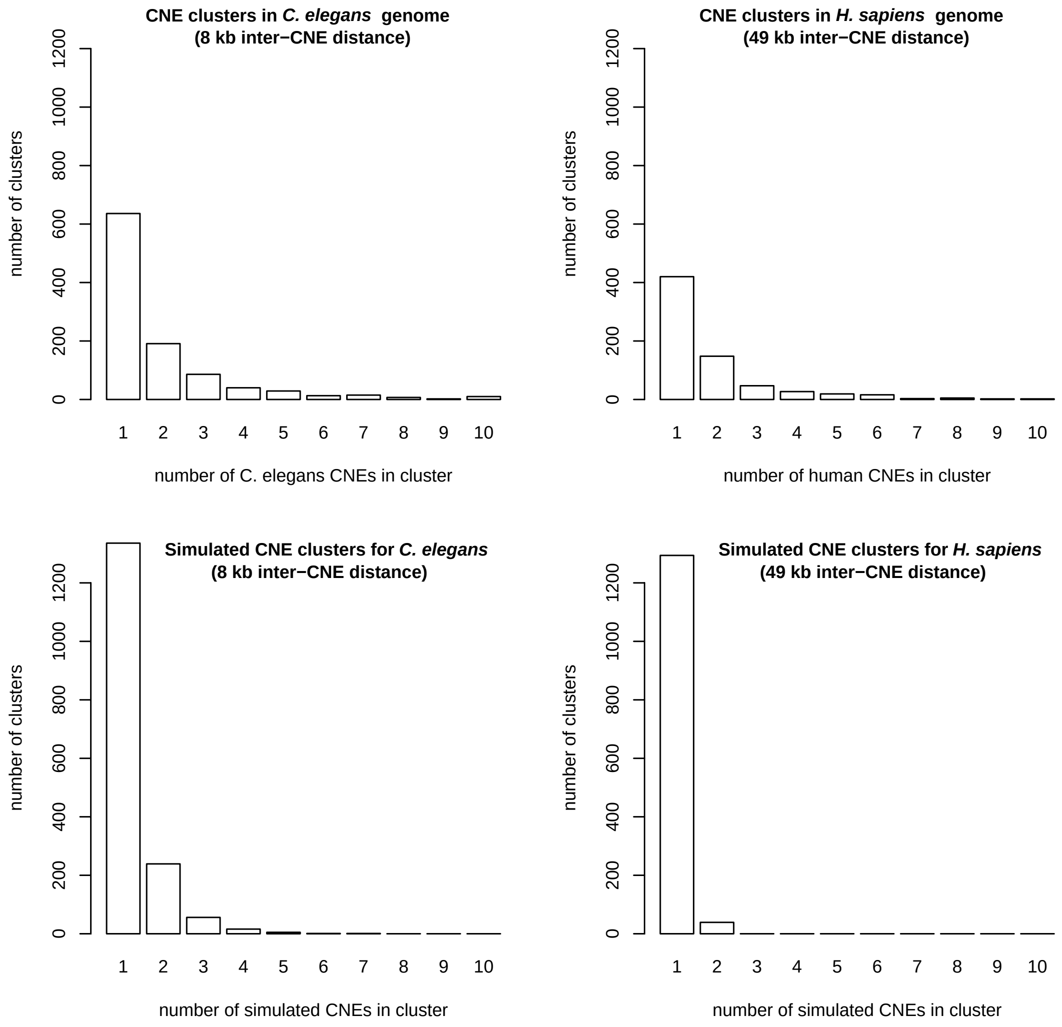

Supplement: Additional data file 2 — Distribution of distances between CNEs in both the C. elegans and the H. sapiens genomes and for randomized CNE locations. [file gb-2007-8-2-r15-S2.pdf]
