# Supplementary material for: Parallel evolution of conserved non-coding elements that target a common set of developmental regulatory genes from worms to humans
Source: Genome Biol. 2007 Feb 2;8(2):R15. doi: 10.1186/gb-2007-8-2-r15 (PMC1852409; doi:10.1186/gb-2007-8-2-r15)

Figure S3. The distribution of distances between intergenic wCNEs and their nearest genes reveals many distal predicted *cis*-regulatory elements in the *C. elegans* genome. For each of the 1,460 intergenic wCNEs, we calculated the distance from the nearest transcription start site of a protein-coding gene.

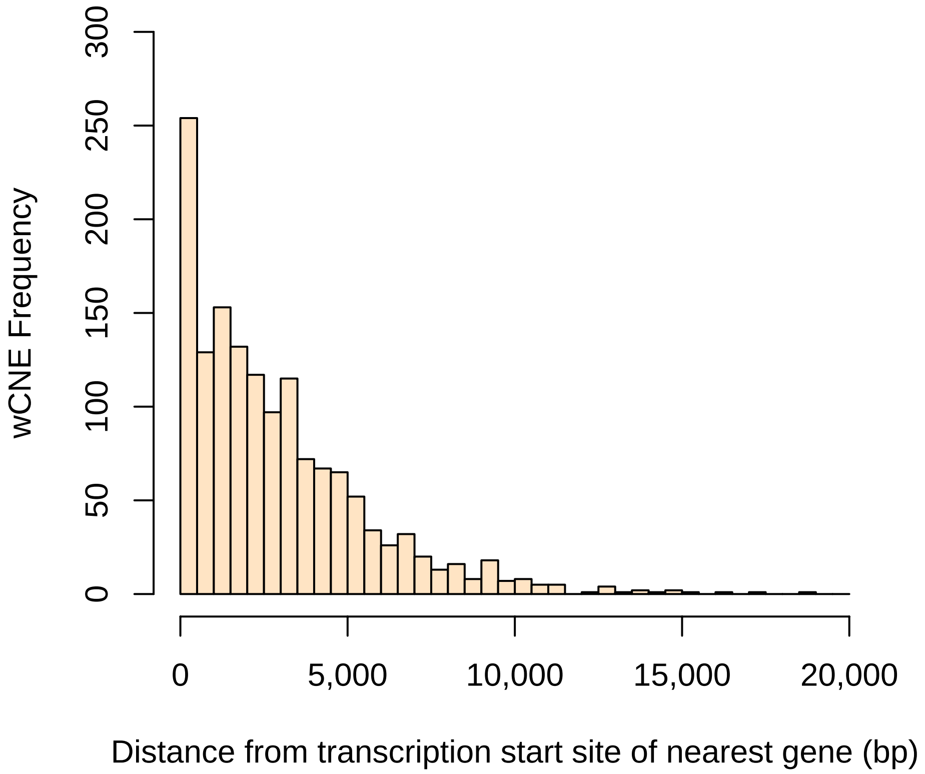

Supplement: Additional data file 3 — Distribution of distances between intergenic wCNEs and their nearest genes. [file gb-2007-8-2-r15-S3.pdf]
