# Supplementary material for: Parallel evolution of conserved non-coding elements that target a common set of developmental regulatory genes from worms to humans
Source: Genome Biol. 2007 Feb 2;8(2):R15. doi: 10.1186/gb-2007-8-2-r15 (PMC1852409; doi:10.1186/gb-2007-8-2-r15)
Supplement: Additional data file 4 — Top-level InterPro protein domains significantly enriched in CNE-associated genes compared to the rest of the genes in the (a) H. sapiens and (b) C. elegans genomes. [file gb-2007-8-2-r15-S4.pdf]

Table S1. Protein domains significantly enriched in CNE-associated genes compared to the rest of the genes in the *H. sapiens* (a) and the *C. elegans* (b) genome. The domains shown in the two tables below constitute all top-level InterPro domains with a positive log odds ratio (LOR) that are significantly associated with CNE-genes and are found in at least 10 genes in either the *H. sapiens* or the *C. elegans* genome. The total number of proteins annotated with at least one InterPro domain was 12,946 in the *C. elegans* genome and 17,801 in the human genome. Protein domain titles in bold are significantly enriched in both CNE-associated gene sets. To account for multiple comparisons, we calculated the p-value threshold at the 5% FDR cut-off using the false discovery rate method by Benjamini and Hochberg [2] (threshold p = 3.6e-3). CI: Confidence interval.

key:
DNA-binding domain
Domain commonly found in DNA-binding proteins
Signalling protein domain
Domain commonly found in signalling proteins
Domain found in both DNA-binding and signalling proteins
Other domain/ domain found in many diverse proteins

(a) InterPro domain	CNE-genes with domain in human	All genes with domain in human	LOR (±CI)	P-value
POU	24	86	3.14 (±0.48)	< 2.2e-16
**Homeodomain-like**	63	323	2.80 (±0.30)	< 2.2e-16
Antifreeze protein, type I	23	176	2.19 (±0.45)	< 2.2e-16
**HMG1/2 (high mobility group) box**	10	69	2.27 (±0.67)	1.22e-011
Paired-like homeodomain protein, OAR	5	15	3.34 (±1.04)	1.48e-010
**Zinc finger, C2H2-type**	38	805	1.09 (±0.34)	2.63e-010
**Eggshell protein**	10	82	2.07 (±0.66)	3.43e-010
Steroid hormone receptor	13	156	1.65 (±0.57)	6.78e-009
Basic helix-loop-helix dimerisation region bHLH	10	109	1.75 (±0.65)	5.68e-008
Ribosomal protein P2	6	42	2.24 (±0.84)	9.30e-008
Vitamin D receptor	6	47	2.11 (±0.83)	3.64e-007
Nuclear hormone receptor, DNA-binding	6	47	2.11 (±0.83)	3.64e-007
Steroid nuclear receptor, ligand-binding	6	49	2.06 (±0.83)	5.97e-007
Prion protein	4	23	2.47 (±1.03)	1.37e-006
**LIM, zinc-binding**	7	74	1.77 (±0.76)	2.58e-006
Pollen allergen Poa pIX/Phl pVI, C-terminal	5	40	2.08 (±0.91)	3.27e-006
Fos transforming protein	3	26	1.99 (±1.13)	2.82e-004
UDENN	2	13	2.31 (±1.37)	4.73e-004

p53-like transcription factor, DNA-binding	2	13	2.31 (±1.37)	4.73e-004
Ricin B lectin	3	31	1.79 (±1.12)	8.41e-004
Cell surface receptor IPT/TIG	3	31	1.79 (±1.12)	8.41e-004
Cytokine receptor class 2	2	15	2.15 (±1.35)	9.40e-004
DDENN	2	15	2.15 (±1.35)	9.40e-004
Involucrin repeat	2	15	2.15 (±1.35)	9.40e-004
DENN	2	16	2.07 (±1.35)	1.27e-003
**Winged helix repressor DNA-binding**	7	139	1.09 (±0.74)	2.02e-003
**Ion transport**	8	173	1.00 (±0.70)	2.43e-003
Proline-rich region	33	1185	0.50 (±0.36)	3.29e-003
Haloacid dehalogenase-like hydrolase	3	40	1.51 (±1.10)	3.62e-003

**(b) InterPro domain**	**wCNE-genes with domain in *C. elegans***	**All genes with domain in *C. elegans***	**LOR (±CI)**	**P-value**
Fibronectin, type III	19	46	2.29 (±0.58)	7.44e-015
Immunoglobulin-like	23	77	1.79 (±0.49)	3.20e-013
**Homeodomain-like**	32	139	1.45 (±0.40)	5.81e-013
Immunoglobulin C2 type	16	45	2.05 (±0.61)	1.91e-011
K+ channel, pore region	22	85	1.59 (±0.49)	6.63e-011
**Ion transport**	18	61	1.77 (±0.55)	1.24e-010
Immunoglobulin subtype	11	29	2.14 (±0.74)	6.66e-009
EGF-like, type 3	21	97	1.36 (±0.48)	1.92e-008
**Winged helix repressor DNA-binding**	16	63	1.56 (±0.56)	2.94e-008
Endoglin/CD105 antigen	12	39	1.82 (±0.67)	5.14e-008
**Zinc finger, C2H2-type**	36	230	0.97 (±0.36)	7.51e-008
EGF-like	30	180	1.04 (±0.40)	1.36e-007
PDZ/DHR/GLGF	16	68	1.46 (±0.56)	1.43e-007
Pleckstrin-like	15	71	1.32 (±0.57)	2.46e-006
Rho GTPase activation protein	8	26	1.82 (±0.82)	5.96e-006
Laminin G	6	15	2.23 (±1.00)	6.48e-006
Cadherin	5	10	2.63 (±1.18)	6.70e-006
Laminin G, Thrombospondin-type, N terminal	6	16	2.12 (±0.98)	1.14e-005
DM DNA-binding	5	11	2.45 (±1.14)	1.24e-005
**LIM, zinc-binding**	8	29	1.67 (±0.80)	2.08e-005
Apple-like	7	23	1.80 (±0.87)	2.21e-005
Sterile alpha motif homology	6	18	1.94 (±0.95)	3.23e-005
RA	5	13	2.16 (±1.07)	4.11e-005
EGF	14	76	1.15 (±0.58)	4.67e-005
K+ channel, two pore	9	41	1.36 (±0.73)	1.17e-004
Aspartic acid and asparagine hydroxylation site	8	34	1.45 (±0.78)	1.21e-004
**HMG1/2 (high mobility group) box**	5	15	1.94 (±1.03)	1.21e-004

Ras GTPase	13	73	1.10 (±0.59)	1.33e-004
C2 calcium/lipid-binding region, CaLB	10	49	1.27 (±0.68)	1.34e-004
Peptidase M12B, ADAM/reprolysin	4	11	2.07 (±1.17)	2.67e-004
Src homology-3	11	61	1.12 (±0.64)	3.32e-004
Band 7 protein	4	12	1.94 (±1.14)	4.61e-004
Thrombospondin, type I	8	39	1.28 (±0.76)	5.03e-004
Spectrin repeat	5	20	1.53 (±0.98)	1.05e-003
Peptidase M, neutral zinc metallopeptidases, zinc-binding site	14	95	0.88 (±0.56)	1.11e-003
Pleckstrin homology-type	5	22	1.40 (±0.96)	2.07e-003
EGF-like, laminin	4	16	1.53 (±1.08)	2.75e-003
**Eggshell protein**	3	10	1.78 (±1.27)	3.02e-003
Actin-binding, actinin-type	3	10	1.78 (±1.27)	3.02e-003
