# Supplementary material for: Parallel evolution of conserved non-coding elements that target a common set of developmental regulatory genes from worms to humans
Source: Genome Biol. 2007 Feb 2;8(2):R15. doi: 10.1186/gb-2007-8-2-r15 (PMC1852409; doi:10.1186/gb-2007-8-2-r15)
Supplement: Additional data file 5 — Enrichment for transcription factors among wCNE-associated genes, using two different collections of predicted transcription factors from the C. elegans genome. [file gb-2007-8-2-r15-S5.pdf]

Table S2. Transcription factors are significantly enriched among genes associated with wCNEs. We have used two high-quality transcription factor prediction databases to annotate wCNE-associated genes. DBD is a database of transcription factors predicted through homology to domains of known sequence-specific DNA-binding transcription factors [1]. The *C. elegans* genes analysed in DBD correspond to WormBase release WS150. wTF2.0 is a compendium of computationally predicted and manually curated transcription factors [2]. The *C. elegans* genes analysed in wTF2.0 correspond to WormBase release WS140. Using both databases we have found that the set of genes associated with wCNEs contains a significantly higher proportion of predicted transcription factors than the rest of the genes in the genome. The total number of genes associated with wCNEs is 1,241.

**Database of predicted transcription factors**	**DBD [1]**	**wTF2.0[2]**
Number of genes coding for transcription factors associated with wCNEs	108	137
Number of genes coding for transcription factors in the *C. elegans* genome	723	934
Total number of genes considered in the *C. elegans* genome	20,053	19,735
Log odds ratio	1.037 (±0.213)	1.014 (±0.191)
P-value	< 2.2e-16	< 2.2e-16

References

1. Kummerfeld SK, Teichmann SA: **DBD: a transcription factor prediction database**. *Nucleic Acids Res* 2006, **34**(Database issue):D74-81.
2. Reece-Hoyes JS, Deplancke B, Shingles J, Grove CA, Hope IA, Walhout AJ: **A compendium of Caenorhabditis elegans regulatory transcription factors: a resource for mapping transcription regulatory networks**. *Genome Biol* 2005, **6**(13):R110.
