# Supplementary material for: Parallel evolution of conserved non-coding elements that target a common set of developmental regulatory genes from worms to humans
Source: Genome Biol. 2007 Feb 2;8(2):R15. doi: 10.1186/gb-2007-8-2-r15 (PMC1852409; doi:10.1186/gb-2007-8-2-r15)
Supplement: Additional data file 6 — wCNEs overlapping known cis-regulatory elements. [file gb-2007-8-2-r15-S6.pdf]

Table S3. Overlap of wCNEs with known *cis*-regulatory elements. The genes shown in this table were extracted from literature searches for dissected *cis*-regulatory elements. For each gene, we show the number of spatially associated wCNEs (plus the additional wCNE associated with *lir-1*), the number of wCNEs that overlap dissected *cis*-elements and the references in which the experiments are described. In the case of *lin-26*, there is one wCNE, which is closer to the *lir-1* transcription start site than *lin-26*, so it was associated with *lir-1* in our dataset, however experiments showed that it is likely to be a *lin-26* distal *cis*-element.

**Gene**	**Number of wCNEs**	**Number of wCNEs that overlap experimentally tested sequences showing *cis*-regulatory activity**	**Ref.**
myo-2	1	1	[1]
lin-26	3 (+ 1 spatially associated with lir-1)	3 (+1)	[2]
ceh-22	2	1 (the other wCNE is intronic)	[3]
ser-2	5	5	[4]
ric-4	1	1	[5]
egl-5	1	0 (wCNE lies downstream)	[6]
ttx-3	0	-	[4]
sra-11	0	-	[4]
kal-1	0	-	[4]
hen-1	0	-	[4]
unc-17	0	-	[4]
ceh-10	0	-	[4]
ceh-23	0	-	[4]
dpy-7	0	-	[7]
egl-17	0	-	[8]
mec-3	0	-	[9]
her-1	0	-	[10]

References

1. Kalb JM, Beaster-Jones L, Fernandez AP, Okkema PG, Goszczynski B, McGhee JD: **Interference between the PHA-4 and PEB-1 transcription factors in formation of the Caenorhabditis elegans pharynx**. *J Mol Biol* 2002, **320**(4):697-704.
2. Landmann F, Quintin S, Labouesse M: **Multiple regulatory elements with spatially and temporally distinct activities control the expression of the epithelial differentiation gene lin-26 in C. elegans**. *Dev Biol* 2004, **265**(2):478-490.
3. Vilimas T, Abraham A, Okkema PG: **An early pharyngeal muscle enhancer from the Caenorhabditis elegans ceh-22 gene is targeted by the Forkhead factor PHA-4**. *Dev Biol* 2004, **266**(2):388-398.
4. Wenick AS, Hobert O: **Genomic cis-regulatory architecture and trans-acting regulators of a single interneuron-specific gene battery in C. elegans.** *Dev Cell* 2004, **6**(6):757-770.
5. Hwang SB, Lee J: **Neuron cell type-specific SNAP-25 expression driven by multiple regulatory elements in the nematode Caenorhabditis elegans**. *J Mol Biol* 2003, **333**(2):237-247.
6. Teng Y, Girard L, Ferreira HB, Sternberg PW, Emmons SW: **Dissection of cis-regulatory elements in the C. elegans Hox gene egl-5 promoter**. *Dev Biol* 2004, **276**(2):476-492.
7. Gilleard JS, Barry JD, Johnstone IL: **cis regulatory requirements for hypodermal cell-specific expression of the Caenorhabditis elegans cuticle collagen gene dpy-7**. *Mol Cell Biol* 1997, **17**(4):2301-2311.
8. Cui M, Han M: **Cis regulatory requirements for vulval cell-specific expression of the Caenorhabditis elegans fibroblast growth factor gene egl-17**. *Dev Biol* 2003, **257**(1):104-116.
9. Xue D, Finney M, Ruvkun G, Chalfie M: **Regulation of the mec-3 gene by the C.elegans homeoproteins UNC-86 and MEC-3**. *Embo J* 1992, **11**(13):4969-4979.
10. Li W, Streit A, Robertson B, Wood WB: **Evidence for multiple promoter elements orchestrating male-specific regulation of the her-1 gene in Caenorhabditis elegans**. *Genetics* 1999, **152**(1):237-248.
