# Supplementary material for: Parallel evolution of conserved non-coding elements that target a common set of developmental regulatory genes from worms to humans
Source: Genome Biol. 2007 Feb 2;8(2):R15. doi: 10.1186/gb-2007-8-2-r15 (PMC1852409; doi:10.1186/gb-2007-8-2-r15)
Supplement: Additional data file 7 — Annotation features from WormBase that were used to annotate wCNEs. [file gb-2007-8-2-r15-S7.pdf]

Table S4. WormBase annotation features used to annotate wCNEs. Conserved elements in *C. elegans* were annotated according to the type of WormBase annotation feature they overlapped (WS140). wCNEs are elements that do not overlap any of the following genomic features or they lie entirely within introns of protein-coding genes and do not overlap "Exon" type of features (indicated by an asterisk). wCNEs were also filtered for repeats and non-coding RNAs.

Brief annotation	WormBase annotation feature		Exon
Protein coding primary transcript	Coding_transcript	protein_coding_primary_transcript	
Other transcribed & repeats	BLAT_NEMATODE	translated_nucleotide_match	*
	Coding_transcript	coding_exon	*
	curated	CDS	
	curated	coding_exon	*
	wublastx	protein_match	*
	BLAT_ncRNA_BEST	nucleotide_match	*
	BLAT_ncRNA_OTHER	nucleotide_match	*
	miRNA	exon	*
	miRNA	miRNA_primary_transcript	
	Non_coding_transcript	exon	*
	Non_coding_transcript	nc_primary_transcript	
	rRNA	exon	*
	rRNA	rRNA_primary_transcript	
	scRNA	exon	*
	scRNA	scRNA_primary_transcript	
	snoRNA	exon	*
	snoRNA	snoRNA_primary_transcript	
	snRNA	exon	*
	snRNA	snRNA_primary_transcript	
	tRNAscan-SE-1.23	exon	*
	tRNAscan-SE-1.23	tRNA_primary_transcript	
	Genefinder	CDS	
	Genefinder	coding_exon	*
	Genefinder	exon	*
	history	CDS	
	history	coding_exon	*
	history	exon	*
	history	Transcript	
	history	Pseudogene	
	Pseudogene	exon	*
	Pseudogene	Pseudogene	
	inverted	inverted_repeat	
	RepeatMasker	repeat_region	
	tandem	tandem_repeat	

	BLAT_EST_BEST	EST_match	*
	BLAT_EST_OTHER	EST_match	*
	BLAT_mRNA_BEST	cDNA_match	*
	BLAT_mRNA_OTHER	cDNA_match	*
	BLAT_OST_BEST	expressed_sequence_match	*
	BLAT_OST_OTHER	expressed_sequence_match	*
	Coding_transcript	exon	*
	Coding_transcript	five_prime_UTR	*
	Coding_transcript	protein_coding_primary_transcript	
	Coding_transcript	three_prime_UTR	*
	Curated	exon	*
	Gene	gene	
	Operon	operon	
	SAGE_transcript	transcript	
	TEC_RED	nucleotide_match	
	BLAT_TC1_BEST	nucleotide_match	
	BLAT_TC1_OTHER	nucleotide_match	
	Transposon	transposable_element	
	Transposon_CDS	coding_exon	*
	Transposon_CDS	exon	*
	Transposon_CDS	transposable_element	
	twinscan	CDS	
	twinscan	coding_exon	*
	twinscan	exon	*
Intron	Coding_transcript	intron	
	Curated	intron	
